# Statin Use and Breast Cancer Survival: A Nationwide Cohort Study from Finland

**DOI:** 10.1371/journal.pone.0110231

**Published:** 2014-10-20

**Authors:** Teemu J. Murtola, Kala Visvanathan, Miia Artama, Harri Vainio, Eero Pukkala

**Affiliations:** 1 University of Tampere, School of Medicine, Tampere, Finland; 2 Tampere University Hospital, Department of Urology, Tampere, Finland; 3 Johns Hopkins Bloomberg School of Public Health, Department of Epidemiology, Baltimore, Maryland, United States of America; 4 Sidney Kimmel Comprehensive Cancer Center, Johns Hopkins School of Medicine, Baltimore Maryland, United States of America; 5 University of Helsinki, Helsinki, Finland; 6 Finnish Institute of Occupational Health, Helsinki, Finland; 7 University of Tampere, School of Health Sciences, Tampere, Finland; 8 The Finnish Cancer Registry, Institute for Statistical and Epidemiological Cancer Research, Helsinki, Finland; University of British Columbia, Canada

## Abstract

Recent studies have suggested that statins, an established drug group in the prevention of cardiovascular mortality, could delay or prevent breast cancer recurrence but the effect on disease-specific mortality remains unclear. We evaluated risk of breast cancer death among statin users in a population-based cohort of breast cancer patients. The study cohort included all newly diagnosed breast cancer patients in Finland during 1995–2003 (31,236 cases), identified from the Finnish Cancer Registry. Information on statin use before and after the diagnosis was obtained from a national prescription database. We used the Cox proportional hazards regression method to estimate mortality among statin users with statin use as time-dependent variable. A total of 4,151 participants had used statins. During the median follow-up of 3.25 years after the diagnosis (range 0.08–9.0 years) 6,011 participants died, of which 3,619 (60.2%) was due to breast cancer. After adjustment for age, tumor characteristics, and treatment selection, both post-diagnostic and pre-diagnostic statin use were associated with lowered risk of breast cancer death (HR 0.46, 95% CI 0.38–0.55 and HR 0.54, 95% CI 0.44–0.67, respectively). The risk decrease by post-diagnostic statin use was likely affected by healthy adherer bias; that is, the greater likelihood of dying cancer patients to discontinue statin use as the association was not clearly dose-dependent and observed already at low-dose/short-term use. The dose- and time-dependence of the survival benefit among pre-diagnostic statin users suggests a possible causal effect that should be evaluated further in a clinical trial testing statins’ effect on survival in breast cancer patients.

## Introduction

Breast cancer is the leading cause of cancer death among women globally. [1] Experimental studies suggest that statins, a well-established group of cholesterol-lowering drugs, may have antitumor properties against this common cancer. [2]–[4].

Statins reduce cholesterol biosynthesis in the liver by inhibiting 3-hydroxy-3-methylglutaryl-coenzyme-A reductase (HMGCR), the rate-limiting enzyme in the mevalonate pathway. In addition to cholesterol, this pathway produces isoprenoids that are critical for regulation of cell growth. [5] The pathway is also essential for tumor promoting effects of oncogene p53. [6] Further, cholesterol is a critical component of intracellular lipid-rafts, which are crucial for intracellular signaling. [7] Thus statins’ anticancer effects have a biologically plausible background.

Despite promising preclinical results, there is no clear association between statin use and breast cancer incidence. [8] However, some recent studies have reported lowered overall cancer mortality among statin users, [9]–[13] including one study that reported decreased breast cancer mortality among pre-diagnostic statin users, [13] and other studies have suggested that statin use is associated with improved recurrence-free survival among breast cancer patients. [14]–[17] Therefore statins may exert a greater effect on cancer progression versus initiation.

We studied the association between statin use and breast cancer mortality among breast cancer patients in a nationwide population-based cohort.

## Materials and Methods

### Data sources

We used the Finnish cancer registry to obtain information on all breast cancer cases diagnosed in Finland from 1995 to 2003. Due to mandatory reporting of all cancer diagnoses made in the Finnish health care units, the Finnish Cancer Registry has good national coverage, including over 99% of cancer cases in Finland. [18] The information on breast cancer cases included the date of diagnosis, tumor stage (local vs. metastatic, available for 92% of cases in our cohort), tumor morphology (available for all cases), initial treatment selection (surgery, radiation therapy, chemotherapy, hormonal therapy or other) and date and cause of death (cancer death vs. death due to other causes). Information on tumor hormone receptor status or screening history was not available. However, the screening participation rate for breast cancer screening in Finland has been reported to be up to 90% [19].

Detailed, individual-level information on usage of cholesterol-lowering drugs between Jan. 1,1995 and Dec. 31, 2003 was obtained from the national prescription database managed by Social Insurance Institution of Finland (SII). Cancer cases were linked to the prescription database using a unique personal identification number.

SII is a governmental agency providing reimbursements to each Finnish citizen for the cost of medicines prescribed by a physician and purchased in outpatient setting. All reimbursed purchases of drugs approved by the SII (most prescription drugs in clinical use) are recorded in the database. [20] The prescription database includes information on date, dosage, package size and number of packages obtained for each reimbursed purchase.

The cholesterol-lowering drugs in clinical use during the study period and recorded by the prescription database were statins (atorvastatin, cerivastatin, fluvastatin, lovastatin, pravastatin, rosuvastatin and simvastatin), fibrates (bezafibrate, clofibrate, fenofibrate and gemfibrozil) and bile-acid binding resins (cholestyramin and cholestipol).

### Identification of the study cohort

All histologically confirmed invasive breast cancer cases diagnosed in Finnish health care units from 1995 to 2003 and recorded in the Finnish Cancer Registry were included in our study cohort, a total of 31,236 cases (31,114 women, 122 men). Men were excluded from this analysis.

### Lipid-lowering drug usage

The status on post-diagnostic statin use was updated prospectively for each year of follow-up since breast cancer diagnosis. The study participant was categorized as statin user only for the years with recorded statin purchases, regardless of the amount. Persons who discontinued prior post-diagnostic statin use were categorized separately as previous users. Cumulative amount (daily doses), duration (years) and intensity (doses/year of usage) of post-diagnostic use were analyzed as prospective time-dependent continuous variables. At discontinuation the cumulative amount/duration/intensity of statin use stayed at the level reached before the usage was stopped.

For prediagnostic use women who were using statins at the year of diagnosis were categorized as current pre-diagnostic users; those who had use the drugs before but had stopped prior to the diagnosis were categorized as previous pre-diagnostic users. Total cumulative amount, duration and intensity of pre-diagnostic statin use were calculated since 1995 up to the year of diagnosis.

The amount of usage was standardized for different statins using the defined daily doses (DDDs) recommended by the World Health Organization (WHO ATC/DDD index database). [21] The DDD is the assumed average maintenance dose per day for a drug used for its main indication in adults. For each year of follow-up, the total milligram amount for each drug was calculated based on all purchases reimbursed that year. Yearly mg amount was divided by the amount corresponding to 1 DDD to obtain the yearly DDDs. Duration of medication use was calculated as the cumulative number of years of follow-up with recorded statin purchases. The total cumulative amount and duration of usage were obtained by adding together yearly DDDs or years with statin purchases from the entire follow-up. Intensity of statin use, i.e. the number of statin doses used per year was calculated by dividing the yearly number of DDDs with years of usage (DDDs/year). The study population was stratified into tertiles (post-diagnostic use) or by median (pre-diagnostic use) of amount, duration and intensity of usage in order to compare long-term/high-dose/high-intensity use with short-term/low-dose/low-intensity usage.

### Statistical analysis

We used Chi-square test (for categorical variables) and Mann-Whitney-U test (for continuous variables) to evaluate statistical significance of the differences in baseline characteristics between medication users and non-users.

Hazard ratios (HRs) and 95% confidence intervals (CIs) for breast-cancer specific and all-cause mortality were estimated using Cox proportional hazards regression, with years since the date of breast cancer diagnosis as the time-metric. Each cohort member contributed person-time from the diagnosis until the date of death, emigration from the country or the end of study period (common closing date December 31^st^, 2003), whichever came first.

Tumor stage at diagnosis, morphology and treatment choice (surgery, radiation therapy, chemotherapy, hormone therapy or other) were included in the regression model as time-independent variables. The proportional hazards assumption was checked for each time-independent variable by including interaction term with follow-up time into the regression model. In each case, the interaction term was not statistically significant, confirming the assumption.

All HRs are calculated using non-users of cholesterol-lowering drugs as the reference group. We performed the analyses separately with an age-adjusted model and a multivariable adjusted model (adjustment for age, tumor stage, morphology and treatment selection). Analyses on non-statin cholesterol-lowering drugs (fibrates and resins) were additionally adjusted for prior statin usage. We report multivariable-adjusted HRs unless otherwise stated.

Survival trends by increasing amount, duration or intensity of statin use were estimated by stratifying the analysis within tertiles of the amount/duration or deciles of intensity of statin usage. P values for trends by amount, duration or intensity of statin use were calculated by including these variables as continuous, time-dependent variables into Cox regression model.

The analyses were repeated separately for pre-diagnostic (statin use occurring before the years of diagnosis) and post-diagnostic statin use.

To address the potential for confounding by indication we evaluated and controlled for each person’s likelihood of being a statin user post-diagnosis we calculated propensity score using logistic regression model with post-diagnostic statin use as the dependent variable and age, tumor stage, morphology, treatment selection and pre-diagnostic statin usage as categorical independent variables. [22] Of these, pre-diagnostic statin use was the strongest predictor of post-diagnostic use. The propensity from each variable was combined to form a total propensity score for statin use. The analysis was stratified by quartiles of the total propensity score to ensure comparable distribution of background characteristics between statin users and non-users.

We evaluated the impact of death due to non-cancer causes on observed breast cancer mortality with a competing risks regression as described by Fine and Gray, [23] using the same model adjustments as for the multivariable adjusted Cox regression model.

All reported p-values are two-sided. IBM SPSS statistics 20 statistical software (Chicago, Illinois, USA) was used for Cox regression analyses and STATA version 12 (StataCorp LP, College Station, Texas, USA) was used for competing risks regression.

## Results

### Population characteristics

Of the participants, 4,151 (13.3% of the cohort) had used statins between 1995 and 2003, while 313 (1% of the cohort) had used fibrates or resins (Table 1). Of the latter 187 (59.7%) had also used statins during the study period. The most commonly used statins were simvastatin (n = 2,031, 48.9% of statin users), atorvastatin (n = 1,507, 36.3%), and fluvastatin (n = 840, 20.2%).

**Table 1:** Baseline population characteristics of all breast cancer cases diagnosed in Finland during 1995–2003.

	Non-users of any cholesterol-lowering drugs	Statin users^a^	Fibrate or resin users^a^
n (%)	26,941 (86.2%)	4,151 (13.3%)	313 (1%)
Median age at diagnosis (yrs)	58	64	65
P-value	Reference	<0.001	<0.001
Age-group^b^			
> = 55 years	15,919 (59.3%)	3,383 (81.5%)	249 (79.6%)
<55 years	10,918 (40.7%)	768 (18.5%)	64 (20.4%)
P-value	Reference	<0.001	<0.001
Deaths; n (% of the subgroup)	5,658 (21.0%)	318 (7.6%)	50 (16.0%)
Breast cancer deaths; n (% of all deaths)	3,434 (60.7%)	166 (52.2%)	27 (54%)
Years of follow-up (median; 95% range)	3.17 (0.08–8.50)	3.83 (0.08–8.67)	3.50 (0.25–8.51)
Stage at diagnosis:			
Local; n (%)	22,747 (84.8%)	3,696 (89.0%)	277 (88.5%)
Metastatic; n (%)	1,899 (7.1%)	152 (3.7%)	11 (3.5%)
Unknown	2,191 (8.2%)	303 (7.3%)	25 (8.0%)
P-value	Reference	<0.001	0.036
Tumor morphology:			
Ductal ca	20,524 (76.2%)	3,252 (78%)	263 (84%)
Lobular ca	4,278 (15.9%)	643 (15.4%)	32 (10.2%)
Other	2,139 (7.9%)	274 (6.6%)	18 (5.8%)
P-value	Reference	0·005	0.002
Treatment selection:			
Any surgery; n (%)	24,908 (92.5%)	3,989 (95.7%)	297 (94.9%)
P-value	Reference	0.003	NS
Any radiation therapy; n (%)	14,474 (53.7%)	2,291 (55.0%)	170 (54.3%)
Chemotherapy	6,367 (23.6%)	628 (15.1%)	51 (16.3%)
P-value	Reference	<0·001	0.012
Hormonal therapy	6,787 (25.2%)	849 (20.4%)	73 (23.3%)
P-value	Reference	<0·001	NS
Other therapy	220 (0.8%)	17 (0.4%)	1 (0.3%)
P-value	Reference	<0.001	NS

a Any pre-diagnostic or post-diagnostic use.

b Age cutoffs selected to reflect menopausal status of the majority of women at breast cancer diagnosis.

In total, 1,801 women (5.8% of all) had used statins before breast cancer diagnosis, while 71 (0.2%) had used fibrates or resins. The usage continued after the diagnosis in 85% and 38% of previous statin and fibrate/resin users, respectively.

A total of 2,350 new users started statin usage post-diagnosis. Of these 1,880 (80%) remained adherent users until the end of follow-up.

During the median follow-up of 3.25 years post-diagnosis 6,011 (19.2%) participants died, of which 3,619 (60.2%) due to breast cancer. The median follow-up did not differ significantly by medication usage. Compared to the non-users, medication users were older and more likely to have localized invasive ductal carcinoma than lobular carcinoma (Table 1). Surgical treatment was more common among statin users. Characteristics of fibrate and resin users were similar to statin users (Table 1).

### Breast cancer survival by post-diagnostic statin use

Compared to the non-users, current post-diagnostic statin users had lowered risk of breast cancer death (multivariable adjusted HR 0.46, 95% CI 0.38–.055), whereas women who had stopped statin use post-diagnosis (previous users) had elevated risk compared to non-users (HR 1.67, 95% CI 1.22–2.27). The risk decrease among current statin users was observed both in localized and metastatic cases at diagnosis (Table 2).

**Table 2:** Risk of breast cancer death by amount, duration and intensity of post-diagnostic statin use compared to non-users in a cohort of all breast cancer cases diagnosed in Finland during 1995–2003.

	Breast cancer mortality									
	Localized					Metastatic				
Statin use	n of cases(non-users/users)	n of deaths(non-users/users)	person-years offollow-up(non-users/users)	HR(95%CI)_age-adjusted_	HR(95%CI)_multivar. adjusted_ a	n of cases(non-users/users)	n of deaths(non-users/users)	person-yearsoffollow-up(non-users/users)	HR (95%CI)_age-adjusted_	HR(95%CI)_multivar. adjusted_ a
Never	23,098/3,455	2,129/105	86,491/14,831	Reference	Reference	1,930/130	1,011/29	3,323/260	Reference	Reference
Current				0.34 (0.27–0.44)	0.35 (0.28–0.45)				0.48 (0.33–0.72)	0.49 (0.33–0.73)
Previous				1.36 (0.97–1.91)	1.44 (1.02–2.02)				1.78 (0.66–4.78)	1.04 (0.38–2.80)
Amount of statin use										
1st tertile(10–322 DDD)	1,138	47	4,361	0.51 (0.38–0.69)	0.54 (0.40–0.72)	60	21	86	0.70 (0.45–1.08)	0.66 (0.42–1.01)
2nd tertile(333–800 DDD)	1,152	32	4,386	0.43 (0.30–0.60)	0.43 (0.31–0.61)	49	6	93	0.36 (0.17–0.76)	0.37 (0.18–0.79)
3rd tertile(801 DDD or more)	1,165	26	6,075	0.41 (0.27–0.61)	0.42 (0.28–0.62)	21	2	82	0.21 (0.03–1.52)	0.24 (0.03–1.74)
Duration of statin use										
1 year	1,126	39	3,810	0.49 (0.35–0.67)	0.51 (0.37–0.70)	80	20	82	0.56 (0.36–0.86)	0.57 (0.37–0.89)
2–3 years	1,382	39	5,513	0.41 (0.30–0.55)	0.42 (0.31–0.57)	39	7	123	0.42 (0.19–0.94)	0.38 (0.17–0.86)
4 years or longer	947	27	5,508	0.52 (0.34–0.77)	0.52 (0.35–0.78)	11	2	55	0.90 (0.22–3.66)	0.73 (0.18–2.98)
										
Intensity of statin use (DDDs/year)										
14–183	1,143	54	4,847	0.59 (0.44–0.78)	0.61 (0.45–0.81)	56	17	114	0.87 (0.53–1.43)	0.66 (0.40–1.09)
184–300	1,208	29	4,985	0.36 (0.25–0.52)	0.36 (0.25–0.53)	35	11	71	0.41 (0.19–0.85)	0.43 (0.20–0.90)
301 or more	1,104	22	4,999	0.42 (0.29–0.60)	0.43 (0.30–0.62)	39	1	75	0.33 (0.15–0.73)	0.42 (0.19–0.94)

a Calculated with Cox regression model adjusted for age, tumor stage and morphology, treatment selection and pre-diagnostic statin use.

DDD = Defined Daily Dose.

**Figure 1:**
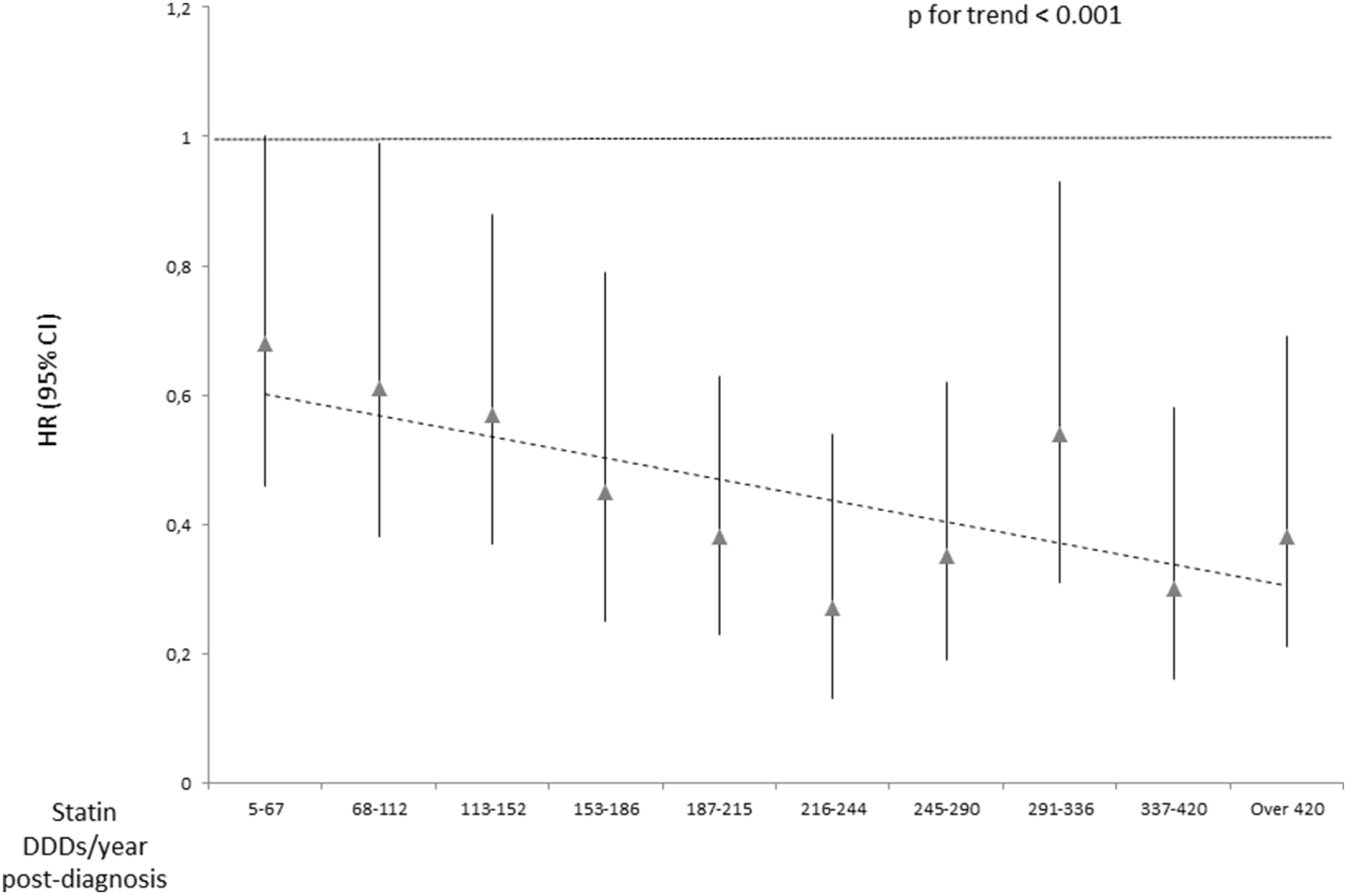
Trend in breast cancer mortality by intensity (doses/year) of post-diagnostic statin use. Nationwide cohort of all female breast cancer patients in Finland during 1995–2003.

In stratified analyses the risk decrease strengthened by increasing cumulative amount and intensity of post-diagnostic use, especially among participants with metastatic tumors (Table 2). No statistically significant trends were observed by increasing amount and duration of post-diagnostic statin use, but a significant decreasing trend in breast cancer mortality was observed with increasing intensity of statin usage (p for trend<0.001) (Figure 1).

### Pre-diagnostic statin use and survival

**Figure 2:**
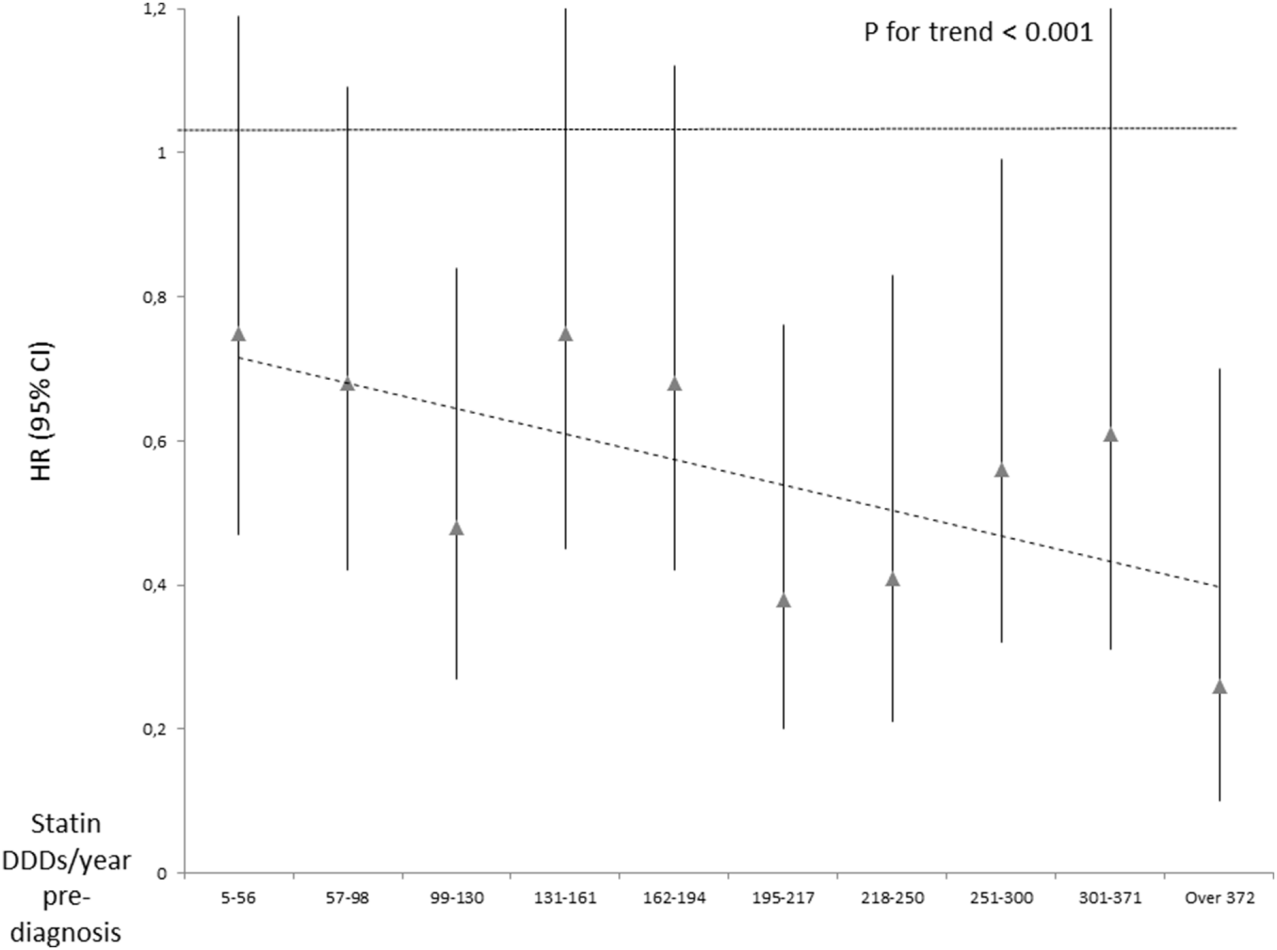
Trend in breast cancer mortality by intensity (doses/year) of pre-diagnostic statin use. Nationwide cohort of all female breast cancer patients in Finland during 1995–2003.

Women who had used statins pre-diagnosis and were still users at the year of breast cancer diagnosis had lowered risk of breast cancer death compared to non-users (HR 0.54, 95% CI 0.44–0.67), whereas previous pre-diagnostic use (usage stopped before the year of diagnosis) was not associated with the risk (HR 0.70, 95% CI 0.46–1.07) (Table 3). A significant decreasing trend by increasing cumulative amount, duration and intensity of pre-diagnostic statin was observed (Table 3). This was observed both for localized and metastatic cases at diagnosis. Unlike for post-diagnostic usage, no significant risk decrease was observed for low-intensity pre-diagnostic use, but only for high-intensity usage (Figure 2).

**Table 3:** Risk of breast cancer death by amount, duration and intensity of statin use pre-diagnosis compared to non-users in a cohort of all breast cancer cases diagnosed in Finland during 1995–2003, with information on medication use available since 1995.

	Breast cancer mortality								
	All cases			Localized			Metastatic		
Prediagnosticstatin use†	n ofcases	n ofdeaths	HR(95% CI) _multivar. adjusted_ *	n ofcases	n ofdeaths	HR(95% CI) _multivar. adjusted_ *	n ofcases	n ofdeaths	HR(95% CI) _multivar. adjusted_ *
None	28,871	3,486	Reference	24,599	2,159	Reference	1,932	1,003	Reference
Current	1,896	96	0.54 (0.44–0.67)	1,652	62	0.60 (0.46–0.77)	106	28	0.58 (0.40–0.84)
Previous	347	22	0.70 (0.46–1.07)	302	13	0.82 (0.48–1.42)	22	9	1.03 (0.53–2.00)
Amount of use‡									
1–495 DDD	1,123	82	0.69 (0.55–0.86)	978	54	0.76 (0.58–0.99)	61	22	0.79 (0.51–1.20)
496 DDD or more	1,120	36	0.40 (0.29–0.56)	976	21	0.44 (0.28–0.67)	67	15	0.51 (0.30–0.86)
P for trend¶			<0.001			<0.001			0.006
Years of use‡									
1–3 years	1,479	95	0.65 (0.53–0.80)	1,286	61	0.69 (0.53–0.89)	85	28	0.77 (0.53–1.12)
4 years or longer	764	23	0.37 (0.25–0.56)	668	14	0.45 (0.27–0.76)	43	9	0.43 (0.22–0.83)
P for trend¶			<0.001			<0.001			0.004
Intensity of use‡									
195 DDDs/yearor less	1,122	78	0.66 (0.53–0.83)	981	48	0.71 (0.53–0.94)	69	24	0.70 (0.47–1.05)
over 196DDDs/years	1,121	40	0.44 (0.32–0.60)	973	27	0.52 (0.36–0.76)	59	13	0.57 (0.33–0.98)
P for trend¶			<0.001			<0.001			0.010

*Calculated with Cox regression model adjusted for age, tumor morphology and treatment selection.

† Women with statin usage at the year of diagnosis categorized as current pre-diagnostic users; women with statin usage before the diagnosis but not at the year of diagnosis considered previous pre-diagnostic users.

‡ Stratum cut-point set at median of amount, duration and intensity of usage.

¶ Calculated by adding total cumulative number of pre-diagnostic DDDs, years of usage or intensity of use (DDDs/year) as a continuous variable into the Cox regression model.

### Stratified analyses

Stratification by quartiles of propensity score ensured similar propensity for post-diagnostic statin use between users and non-users in the first two quartiles (standardized mean difference 0.09 and 0.05, respectively). In the third and fourth quartiles the propensity score was not perfectly balanced between statin users and non-users (standardized mean difference 0.117 and 1.22, respectively), mainly because post-diagnostic statin users in these quartiles were mostly also pre-diagnostic users, which was the strongest predictor of post-diagnostic use causing high propensity scores.

For post-diagnostic statin use the risk of breast cancer death was similarly decreased in all quartiles of propensity score (Table 4). Further, stratification by pre-diagnostic statin use, age group or treatment selection did not clearly modify the risk decrease (Table 4).

**Table 4:** Risk of breast cancer death by current pre-diagnostic and post-diagnostic statin use within a cohort of all breast cancer patients diagnosed in Finland during 1995–2003.

	Risk of breast cancer death	
	Pre-diagnostic statin use	Post-diagnostic statin use
	HR (95% CI) _multivar. adjusted_ a	HR (95% CI) _multivar. adjusted_ a
Propensity score^b^:		
1st quartile	1.23 (0.66–2.29)	0.42 (0.24–0.74)
2nd quartile	0.82 (0.39–1.73)	0.26 (0.12–0.59)
3rd quartile	0.79 (0.43–1.48)	0.51 (0.33–0.78)
4th quartile	0.84 (0.65–1.10)	0.50 (0.38–0.66)
Age		
> = 55 years	0.73 (0.39–1.36)	0.44 (0.26–0.75)
<55 years	0.59 (0.47–0.72)	0.39 (0.31–0.48)
Pre-diagnostic statin use		
Yes	-	0.45 (0.27–0.75)
No Initial treatment choice	-	0.31 (0.22–0.44)
Surgery:		
Yes	0.63 (0.48–0.83)	0.39 (0.30–0.50)
No	0.63 (0.37–1.07)	0.49 (0.26–0.88)
Radiation therapy:		
Yes	0.68 (0.46–1.00)	0.30 (0.20–0.44)
No	0.45 (0.32–0.63)	0.38 (0.28–0.52)
Chemotherapy:		
Yes	0.75 (0.50–1.13)	0.45 (0.29–0.69)
No	0.48 (0.36–0.64)	0.38 (0.30–0.49)
Hormone therapy:		
Yes	0.70 (0.47–1.05)	0.53 (0.36–0.79)
No	0.47 (0.35–0.62)	0.33 (0.25–0.43)
Combination treatments:		
Surgery and radiation therapy	0.71 (0.54–0.93)	0.37 (0.28–0.49)
Surgery and chemotherapy	1.06 (0.76–1.47)	0.45 (0.31–0.64)
Surgery and hormone therapy	0.77 (0.54–1.09)	0.47 (0.33–0.67)
Radiation and chemotherapy	1.11 (0.78–1.57)	0.45 (0.31–0.66)
Radiation and hormone therapy	0.89 (0.63–1.28)	0.54 (0.37–0.78)

Analysis stratified by propensity for post-diagnostic statin use, population characteristics at baseline and primary treatment selection.

a Calculated with Cox regression model adjusted for age, tumor stage and morphology and treatment selection.

b Propensity for post-diagnostic statin usage as a function of age, tumor stage and morphology, initial treatment choice and pre-diagnostic statin use.

The risk estimates for pre-diagnostic statin use were mostly non-significantly decreased in stratified analysis, with no clear effect modification (Table 4).

### All-cause mortality

Similar to breast cancer-specific mortality also all-cause mortality was lowered in current, but not previous pre- and post-diagnostic statin users. Again, the association was clearer with continued and more intensive usage (Table 5).

**Table 5:** Overall risk of death among post-diagnostic and pre-diagnostic statin users compared to non-users.

	Overall risk of death					
	Localized cases at diagnosis			Metastatic cases at diagnosis		
	n of cases	n of deaths	HR (95% CI) _multivar. adjusted_ *	n of cases	n of deaths	HR (95% CI) _multivar. adjusted_ *
Post-diagnosticstatin use	23,098/3,455(non-users/users)	3,779/232(non-users/users)		1,930/130(non-users/users)	1,154/38(non-users/users)	
None			Ref			Ref
Current			0.39 (0.33–0.46)			0.55 (0.39–0.78)
Previous			1.27 (0.98–1.65)			1.16 (0.48–2.82)
Amount of statin use						
1st tertile(10–322 DDD)	1,138	91	0.56 (0.45–0.69)	60	26	0.73 (0.49–1.08)
2nd tertile(333–800 DDD)	1,152	73	0.48 (0.38–0.61)	49	9	0.41 (0.21–0.80)
3rd tertile(801 DDD or more)	1,165	44	0.37 (0.27–0.50)	21	3	0.38 (0.09–1.53)
Duration ofstatin use						
1 year	1,126	78	0.55 (0.44–0.69)	80	26	0.63 (0.43–0.93)
2–3 years	1,382	88	0.46 (0.37–0.57)	39	10	0.50 (0.26–0.96)
4 years orlonger	947	42	0.41 (0.30–0.57)	11	2	0.58 (0.14–2.36)
Intensity of statin use (DDDs/year)						
14–183	1,143	102	0.59 (0.47–0.73)	56	23	0.79 (0.51–1.22)
184–300	1,208	66	0.41 (0.32–0.53)	35	12	0.42 (0.21–0.84)
301 or more	1,104	40	0.45 (0.34–0.58)	39	3	0.47 (0.23–0.94)
						
**Pre-diagnostic** **statin use**	**n of cases**	**n of deaths**	**HR (95% CI)** **_multivar. adjusted_** *	**n of cases**	**n of deaths**	**HR (95% CI)** **_multivar. adjusted_** *
None	24,599	3,841	Ref	1,932	1,144	Ref
Current	1,652	120	0.58 (0.49–0.70)	106	36	0.66 (0.47–0.92)
Previous	302	26	0.80 (0.54–1.17)	22	12	1.18 (0.67–2.10)
Amount of use						
1–495 DDD	978	94	0.69 (0.56–0.84)	61	29	0.91 (0.63–1.31)
496 DDD or more	976	52	0.51 (0.39–0.68)	67	19	0.58 (0.37–0.92)
Years of use						
1–3 years	1,286	114	0.66 (0.55–0.80)	85	35	0.84 (0.60–1.17)
4 yearsor longer	668	32	0.49 (0.34–0.69)	43	13	0.57 (0.33–0.99)
Intensityof use						
195DDDs/year or less	981	89	0.68 (0.55–0.83)	69	32	0.82 (0.57–1.17)
Over 196DDDs/years	973	57	0.54 (0.41–0.70)	59	16	0.63 (0.38–1.03)

Cohort of all breast cancer patients diagnosed in Finland during 1995–2003.

*Calculated with Cox regression model adjusted for age, tumor stage and morphology and treatment selection.

DDD = Defined Daily Dose.

### Survival by statin type

All three most commonly used statins were associated with decreased risk of breast cancer death in participants with localized tumors both when used pre- or post-diagnosis (Table 6). Also post-diagnostic use of hydrophilic pravastatin was linked with lowered risk, whereas pre-diagnostic use was not. Among participants with metastatic disease at diagnosis, only simvastatin users had decreased risk of breast cancer death. However, the lack of significant associations for other statins may be due to small numbers. (Table 6).

**Table 6:** Breast cancer-specific and overall mortality by current pre-diagnostic and post-diagnostic use of specific statins.

	Breast cancer mortality						All-cause mortality			
Statin type*	Localized			Metastatic			Localized		Metastatic	
	n ofcases	n ofdeaths	HR (95% CI)_multivar. adjusted_ †	n ofcases	n ofdeaths	HR (95% CI)_multivar. adjusted_ †	n ofdeaths	HR (95% CI)_multivar. adjusted_ †	n ofdeaths	HR (95% CI)_multivar. adjusted_ †
**No post-diagnostic** **statin use**	23,098	2,129	Reference	1,930	1,011	Reference	3,779	Reference	1,154	Reference
**Simvastatin**										
Post-diagnostic use	1,612	45	0.40 (0.28–0.57)	50	8	0.31 (0.14–0.69)	94	0.43 (0.33–0.55)	13	0.46 (0.25–0.86)
Pre-diagnostic use	693	28	0.67 (0.46–0.98)	41	8	0.36 (0.18–0.73)	55	0.66 (0.51–0.87)	13	0.55 (0.32–0.95)
**Atorvastatin**										
Post-diagnostic use	1,241	20	0.23 (0.13–0.40)	47	9	0.68 (0.34–1.36)	46	0.35 (0.25–0.48)	11	0.66 (0.34–1.27)
Pre-diagnostic use	420	7	0.42 (0.20–0.87)	35	9	1.00 (0.12–8.61)	17	0.52 (0.32–0.83)	10	1.00 (0.14–6.95)
**Fluvastatin**										
Post-diagnostic use	548	22	0.33 (0.17–0.63)	17	5	0.53 (0.20–1.44)	39	0.31 (0.19–0.50)	5	0.45 (0.17–1.23)
Pre-diagnostic use	246	10	0.57 (0.30–1.05)	12	4	0.73 (0.27–1.99)	16	0.46 (0.28–0.75)	4	0.61 (0.22–1.66)
**Pravastatin**										
Post-diagnostic use	374	14	0.45 (0.21–0.94)	10	1	-	22	0.44 (0.25–0.76)	2	0.41 (0.10–1.63)
Pre-diagnostic use	121	9	1.20 (0.62–2.32)	6	0	-	13	0.92 (0.53–1.58)	1	0.35 (0.05–2.52)

Cohort of all breast cancer patients diagnosed in Finland during 1995–2003.

*Statin types are not mutually exclusive, i.e. person who has used two types of statins (e.g. atorvastatin and simvastatin) is counted as a user in both categories.

† Calculated with Cox regression model adjusted for age, tumor stage and morphology, treatment selection and pre-diagnostic statin use.

### Sensitivity analyses

Fibrates and bile-acid binding resins were not associated with all-cause mortality in patients with localized cancer (HR 1.06, 95% CI 0.47–2.37) but were linked to higher mortality in patients with metastatic disease (HR 1.85, 95% CI 1.07–3.20). The number of fibrate/resin users was too low to estimate breast cancer-specific mortality.

Decreased risk of breast cancer death among statin users was not explained by increased risk of death from other causes; the mortality decrease was observed also in multivariable adjusted competing risks regression, with non-cancer deaths as a competing cause of death (HR 0.27, 95% CI 0.22–0.32).

Women dying of breast cancer may have been more likely to drop statin use during the final months of life. However, the inverse association between post-diagnostic statin use and breast cancer mortality remained after exclusion of changes to statin usage status during the final year of follow-up (HR 0.33, 95% CI 0.24–0.45 and 0.63, 95% CI 0.39–1.02 for localized and metastatic cancer, respectively).

The impact of prevalent user bias was evaluated by limiting the analysis to new post-diagnostic statin users only. The risk decrease was observed also in this group of statin users, but with no dose-dependence by amount, duration or intensity of use (Table 7). However, after limiting the analysis to adherent new post-diagnostic users, a significant decreasing trend in breast cancer deaths was observed by years and intensity (p for trend = 0.018 and 0.006, respectively) but not by cumulative amount of post-diagnostic use.

**Table 7:** Risk of breast cancer death by amount, years and intensity of post-diagnostic statin use as compared to non-users.

	Risk of breast cancer death		
Statin use	n of cases	n of deaths	HR (95% CI) _multivar. adjusted_ a
Never	26,963/1,908(users/non-users)	3,439/47(users/non-users)	Reference
Current			0.31 (0.22–0.44)
Former			1.04 (0.60–1.80)
	**Risk of breast cancer death by amount of post-Dx statin use**		
Amount of statin use			
1st tertile (10–322 DDD)	755	25	0.41 (0.27–0.61)
2nd tertile (333–800 DDD)	600	13	0.32 (0.18–0.56)
3rd tertile (801 DDD or more)	553	9	0.45 (0.23–0.86)
	**Risk of breast cancer death by years of post-Dx statin use**		
Years of statin use			
1 year	687	23	0.41 (0.27–0.62)
2–3 years	760	16	0.35 (0.22–0.58)
4 years or longer	461	8	0.41 (0.19–0.86)
	**Risk of breast cancer death by intensity of post-Dx statin use**		
Intensity of statin use			
14–183 DDDs/year	773	25	0.44 (0.29–0.67)
184–300 DDDs/year	632	13	0.28 (0.15–0.52)
301 DDDs/year or more	503	9	0.42 (0.24–0.74)

Statin users limited to new post-diagnostic users only.

a Calculated with Cox regression model adjusted for age, tumor stage and morphology and treatment selection.

DDD = Defined Daily Dose.

Hazard of breast cancer death remained decreased among post-diagnostic statin users when the minimum follow-up was set to be 5 or 7 years (HR 0.23, 95% CI 0.13–0.39 and HR 0.42, 95% CI 0.19–0.95, respectively), suggesting that the risk decrease remains even in the long-term.

## Discussion

We have demonstrated lowered risk of breast cancer death among statin users in a nationwide cohort of all breast cancer patients diagnosed in Finland during a period of nine years. The risk decrease was observed for both localized and metastatic disease at diagnosis, and both for pre-diagnostic and post-diagnostic statin use. The association was dose-dependent especially for pre-diagnostic usage. The risk decrease was not modified by differences in age, tumor characteristics and treatment selection between statin users and non-users. This association was not observed for other types of cholesterol-lowering drugs despite similar age, tumor and treatment characteristics, and was not explained by competing causes of death or decreased likelihood of statin usage at the end of life.

Our results could have been affected by healthy user bias, created by a tendency of healthier patients’ greater likelihood to initiate and adhere to statin therapy, leading to decreased likelihood of outcomes not causally related to statin use, such as risk of accidents. [24] In case of cancer mortality this would mean that healthier cancer patients are more likely to initiate statin use, while less healthier would be less likely to initiate usage and more likely to stop previous use. Indeed, when analyzing current and previous post-diagnostic statin use the risk of death was elevated in women who had stopped previous statin use after the diagnosis. Thus post-diagnostic use was likely affected by the healthy adherer bias, i.e. by increased likelihood of fatally ill cancer patients to stop statin usage and lowered likelihood to start it which makes survival in statin users seem better than it really is. This is likely the reason for absence of clear dose-dependence for post-diagnostic statin use, the risk decrease being observed already at short-term and low-dose usage as well as with longer-term usage. However, for pre-diagnostic statin use the risk association was dependent on the amount, duration and intensity as well as timing of statin use, as would be expected in a causal association. As breast cancer could not have affected the patients’ decisions on statin use before the diagnosis, the healthy adherer effect is unlikely to affect pre-diagnostic statin use.

A major strength of our study is the nationwide coverage of all incident breast cancer patients in Finland from 1995–2003, reducing the possibility of selection bias and allowing us several unique opportunities: the ability to evaluate the association by stage, perform analysis by statin type and compare mortality by the type of cholesterol-lowering drug being used. Another important strength is our detailed knowledge on timing, dosage and duration of statin use, allowing incorporation of the time varying nature of the medication use into analysis and reliable estimation of dose-dependence. Although our median follow-up was only 3.25 years post-diagnosis, the results were unchanged in sensitivity analysis with minimum follow-up set to seven years, showing that the mortality decrease remains also in the long-term.

Previous laboratory studies have demonstrated that statins inhibit breast cancer cell growth *in vitro*, [2]–[4] providing biological plausibility to statins’ inhibitory effect on breast cancer progression. A pre-surgical clinical trial supported this by demonstrating decreased proliferation activity and increased apoptosis in high-grade, but not low-grade breast cancer tissue among patients randomized to receive either high-dose fluvastatin (80 mg/day) or low-dose fluvastatin (20 mg/day) for 3–6 weeks before mastectomy. [25] Another presurgical clinical study reported antiproliferative effect of atorvastatin on invasive breast cancer when given for two weeks before mastectomy at 80 mg/day dose. [26] This effect was observed only in tumors expressing HMGCR at baseline, suggesting that statins target this enzyme in breast cancer tissue. Another possible mechanism for the anti-cancer action is decreased estrone sulfate level. [27] Our results are consistent with previous studies reporting lowered overall cancer mortality in statin users. [9]–[13], [17] Similar to our study, one study estimating effects of pre-diagnostic statin use reported lowered breast cancer mortality in a sub-analysis. [13] Another cohort study found no association between breast cancer mortality and self-reported lipid-lowering drug usage at diagnosis. [17] The results of this study could have been biased towards the null as it did not take into account post-diagnostic statin use. Our study is the largest study to examine this question with ability to analyze statin usage occurring both before and after breast cancer diagnosis.

Cardiovascular disease prevention trials have shown lowered overall mortality in statin users compared to the non-users. [28] A recent meta-analysis of such trials concluded that lowering LDL with statins did not affect cancer risk or mortality during median follow-up of 4.8 years. [29] However, due to the inclusion criteria of included trials most participants did not have cancer at the baseline. Because 5-year disease-specific survival in breast cancer is up to 89% [30] the risk of dying of breast cancer within the next 4.8 years in a cohort of cancer-free people at baseline is very low. Therefore the present clinical trials testing statins for prevention of cardiovascular outcomes have too short follow-up to study breast cancer mortality leading to underpowered analysis. In the meta-analysis 41 breast cancer deaths occurred among 85,683 women included in the trials, which translates to 0.96 deaths/10,000 women/year, which is lower than the average number of breast cancer deaths in the general population: 2.26 deaths/10,000 women/year. [30] This demonstrates how selected the participants of the cardiovascular disease prevention trials are in this regard, and the value of population-based studies such as ours. The final proof of statins’ anticancer effects or the lack of such needs to come from clinical trials recruiting specifically cancer patients.

The significant mortality decrease in our study was evident already after short-term post-diagnostic statin use. Spontaneous decrease in serum cholesterol has been reported for years before cancer death. [31] Thus lower mortality observed already at the initiation of usage may have been because people dying of breast cancer had less hypercholesterolemia, i.e. indication for statin use. Nevertheless, statins may also have a direct short-term effect on cancer progression as recent clinical trials have demonstrated decreased breast cancer proliferation after just weeks of statin usage. [25], [26] Whatever the reason for the initial mortality benefit between statin users and non-users, the dose-dependent decrease in breast cancer mortality by increasing intensity of usage supports a causal effect.

When analyzed separately the risk decrease was not clearly modified by statin potency, as similar risk decrease was observed for high-potent atorvastatin as for other statins. Also hydrophilic pravastatin was associated with a similar decrease as lipophilic statins. This suggests that statins’ anticancer effects *in vivo* are due to a systemic effect common to all statins, such as cholesterol-lowering.

Our study has several limitations. We could not evaluate whether statins’ effect on mortality was modified by tumor hormone receptor status as this information was unavailable. Neither did we have information on breast cancer screening history, which could have been more common among statin users, [32] possibly causing lead-time bias by earlier breast cancer diagnoses. However, the observed risk decrease even among metastatic cases at diagnosis indicates that lead-time bias may not affect our results to any great degree. Our data lacked information on life-style factors, such as obesity, and usage of medications apart from cholesterol-lowering drugs. It could be assumed that fibrate and resin users are in general similar to statin users regarding these unmeasured factors, yet lower mortality was observed only in statin users. Thus lifestyle factors may not have a great influence on our results. Finally, we did not have information on serum cholesterol levels in our cohort and could not assess the indications for statin usage.

In conclusion, statin users had lower risk of breast cancer death compared to non-users in a nationwide cohort of Finnish breast cancer patients. Combined with previous evidence from *in vitro* [2]–[4], epidemiological [14]–[17] and clinical studies [25], [26] our study suggests that, apart from cardiovascular benefits, statins may have beneficial effect against breast cancer progression. However, because uncertainty remains due to biases related to differing likelihood for statin use in different patient groups our results need to be confirmed in a randomized clinical trial before statins can be recommended for breast cancer treatment.
